# Morphological analysis of the alveolar bone of the anterior teeth in severe high-angle skeletal Class II and Class III malocclusions assessed with cone-beam computed tomography

**DOI:** 10.1371/journal.pone.0210461

**Published:** 2019-03-25

**Authors:** Jing Ma, Jing Huang, Jiu-hui Jiang

**Affiliations:** 1 Department of Orthodontics, Peking University School and Hospital of Stomatology, Beijing, China; 2 Department of Orthodontics, Beijing Chongwen Hospital of Stomatology, Beijing, China; Augusta University, UNITED STATES

## Abstract

**Objective:**

This Cross-sectional study used cone-beam computed tomography (CBCT) to evaluate the difference in the alveolar bone of the anterior teeth between high-angle adults with severe skeletal Class II malocclusions and Class III malocclusions.

**Materials and methods:**

The CBCT archives from 62 high-angle adults were selected from patients of the Stomatology Hospital of Peking University between October 2017 to January 2018. The 62 high-angle adult subjects were divided into the following 2 groups based on their sagittal jaw relationships: severe skeletal Class II and severe skeletal Class III. Vertical bone level (VBL), alveolar bone area (ABA), and thickness of alveolar bone were measured at 2 mm, 4 mm, and 6 mm below and above to the cemento-enamel junction (CEJ) level, as well as at the apical level. Then, independent samples t-test were conducted for statistical comparisons.

**Results:**

In the maxillary incisors, the labial VBL was smaller in the patients in skeletal Class III group than those in skeletal Class II group (P<0.05). On the labial side, the ABA was significantly thinner in patients in skeletal Class II group than those in skeletal Class III group, especially in terms of the maxillary central incisors’ ABA at 4 mm and 6 mm above the CEJ level (P<0.05), in terms of apical ABA and total ABA of the maxillary lateral incisors (P<0.05). The alveolar bone thickness around maxillary lateral incisors was significantly thinner in patients of skeletal Class II than that of patients of skeletal Class III, especially regarding the apical level on the labial side (P<0.05). The ABA of the mandibular alveolar bone in the area of the lower anterior teeth was significantly thinner in patients in skeletal Class III group than those in skeletal Class II group, especially in terms of apical ABA, total ABA on the labial and lingual sides, and ABA at 6 mm below the CEJ level on the lingual side (P<0.05). In the mandibular lateral incisors, the alveolar bone thickness was significantly thinner in patients in skeletal Class III group than it was in patients in skeletal Class II group, especially regarding the apical level on the lingual side (P<0.05).

**Conclusions:**

The ABA and the alveolar bone thickness of the mandibular anterior teeth were significantly thinner in the severe high-angle group of skeletal Class III adult patients than in the sample of severe high-angle skeletal Class II adult cases. Our study firstly revealed that the roots of the maxillary central and lateral incisors were placed more labially in the subjects of severe high-angle skeletal Class II than in those of severe high-angle skeletal Class III, especially in the lateral incisors.

## Introduction

Skeletal Class II and skeletal Class III malocclusions, which affect the patient’s facial appearance, masticatory function and mental health, are the most common malocclusions in orthodontic patients. A delineation of the limits of orthodontic tooth movement prior to the start of treatment would be extremely beneficial whether using orthodontic therapy alone or using a combination of orthodontic and orthognathic therapy. During teeth movement, alveolar bone remodeling is affected by the orthodontic force, the morphology of alveolar bone and the balance of the muscles of lip and tongue [1,2]. Excessive retraction or proclination of the anterior teeth may result in iatrogenic sequelae, such as root absorption, alveolar bone loss, dehiscence, fenestration, and gingival recession [3,4]. Therefore, morphometric evaluation of alveolar bone of anterior teeth might be a good model to explain the therapeutic limitation of orthodontic tooth movement.

In recent years, the advent of cone-beam computed tomography (CBCT) has allowed more extensive studies evaluating alveolar bone morphology in the anterior region. CBCT has been found to be valuable in that it is more accurate for assessing bony architecture or quantifying bone volume than traditional radiographic images such as panoramic or periapical views [5,6].

Several studies had demonstrated that the morphological features of alveolar bone were affected by vertical and sagittal facial type. Handelman[2] found that the lingual bone level of mandibular incisor apex was wider in Class I and Class II groups than in the Class III group. Mais et al [7] proved that there is a significant relationship between facial type and alveolar bone thickness and height. To the authors’ knowledge, few studies have scientifically assessed both maxillary and mandibular alveolar bone status of high-angle adults with severe skeletal Class II and Class III malocclusions. Our study aimed to use cross-sectional study with cone-beam computed tomography (CBCT) to evaluate the difference in the alveolar bone of the anterior teeth between high-angle adults with severe skeletal Class II malocclusions and Class III malocclusions. Subjects of our study were patients of severe skeletal class II and class III malocclusions with high angle (normal need Orthodontic—orthognathic combination therapy). Furthermore, this study provides an objective basis for the formulation of a clinical program.

## Methods

This is a retrospective study. It is a measurement study of the results of CBCT examination performed before the patients undergo routine clinical treatment in this hospital. It does not have any adverse effects on patients. The experimental program and the exemption from the informed consent application were approved by Peking University Biomedical Ethics Committee(PKUSSIRB-201734035) before the relevant experiments started. One investigator(JH) conducted the selection of participants individually, another author(JM) completed the collection of data and was blinded to participants’ information. The acquired data were analyzed by the third author(JHJ) separately and also blinded to information that could identify individual participants.

### Sample size

According to the results of a preliminary experiment, the total alveolar bone area(ABA) of the labial side for the two groups was 7.5 mm^2^ (high-angle skeletal Class II group) and 3.5 mm^2^ (high-angle skeletal Class III group). The estimation of sample size, alpha = 0.05, beta = 0.8, is approximately 28 cases for each group, as determined by PASS software (NCSS-PASS 11.0.7).

### Study population

The patients were selected from the Stomatology Hospital of Peking University between October 2017 and January 2018. Sixty-two patients were selected according to the following inclusion criteria: (1) adult, male>18 years old, female>16 years old (2) SN / MP≥40°, (3) ANB≥4° or ANB≤-1°. The illustrative information was presented in S1 Fig in supporting information. The exclusion criteria were (1) history of orthodontic treatment, (2) periodontal disease in the anterior region, (3) defective dentition or supernumerary teeth in the anterior region, (4) obvious pathology (cyst or tumor in the alveolar process), (5) facial asymmetry deformity. Sixty-two high-angle patients were divided into the following 2 groups based on their sagittal jaw relationships: Skeletal Class II (21 female, 10 male; mean age,22.8 years) and Skeletal Class III (20 female, 11 male; mean age,21.1years). The basic information and cephalometric characteristics of the subjects are shown in Table 1. A final sample of 62 patients with 702 anterior teeth was selected (Table 2). Forty-two teeth were excluded because of periapical diseases or root absorption.

**Table 1:** Distribution of samples.

Groups	Total, n	Age, n (years)	ANB, n (degrees)	SN-MP, n (degrees)
Skeletal Class II	31	22.8±4.2	7.4±2.5	45.6±4.2
Skeletal Class III	31	21.1±4.9	-3.9±2.5	43.7±4.0

**Table 2:** Distribution of teeth.

Items	Skeletal Class II, n	Skeletal Class III, n	Total, n
Maxillary central incisor (U1)	62	59	121
Maxillary lateral incisor (U2)	62	57	119
Mandibular central incisor (L1)	60	58	118
Mandibular lateral incisor (L2)	56	54	110
Total	240	228	468

### Research procedures

CBCT images were taken with a NewTom VG CBCT machine (Aperio Services, Verona, Italy). The scans were taken in a single 360-rotation at a scan time of 18 seconds, exposure time of 5.4 seconds, 110 kVp, 0.125-mm voxel size, and 150×150 mm field of view. Digital Imaging and Communications in Medicine(DICOM)Raw data were reconstructed into 3-dimensional volumes and saved as DICOM files using the software from the manufacturer. Then, the DICOM files were imported into Dolphin 3D Imaging software (Dolphin Imaging and Management Solutions, Chatsworth, CA, USA).

**Fig 1:**
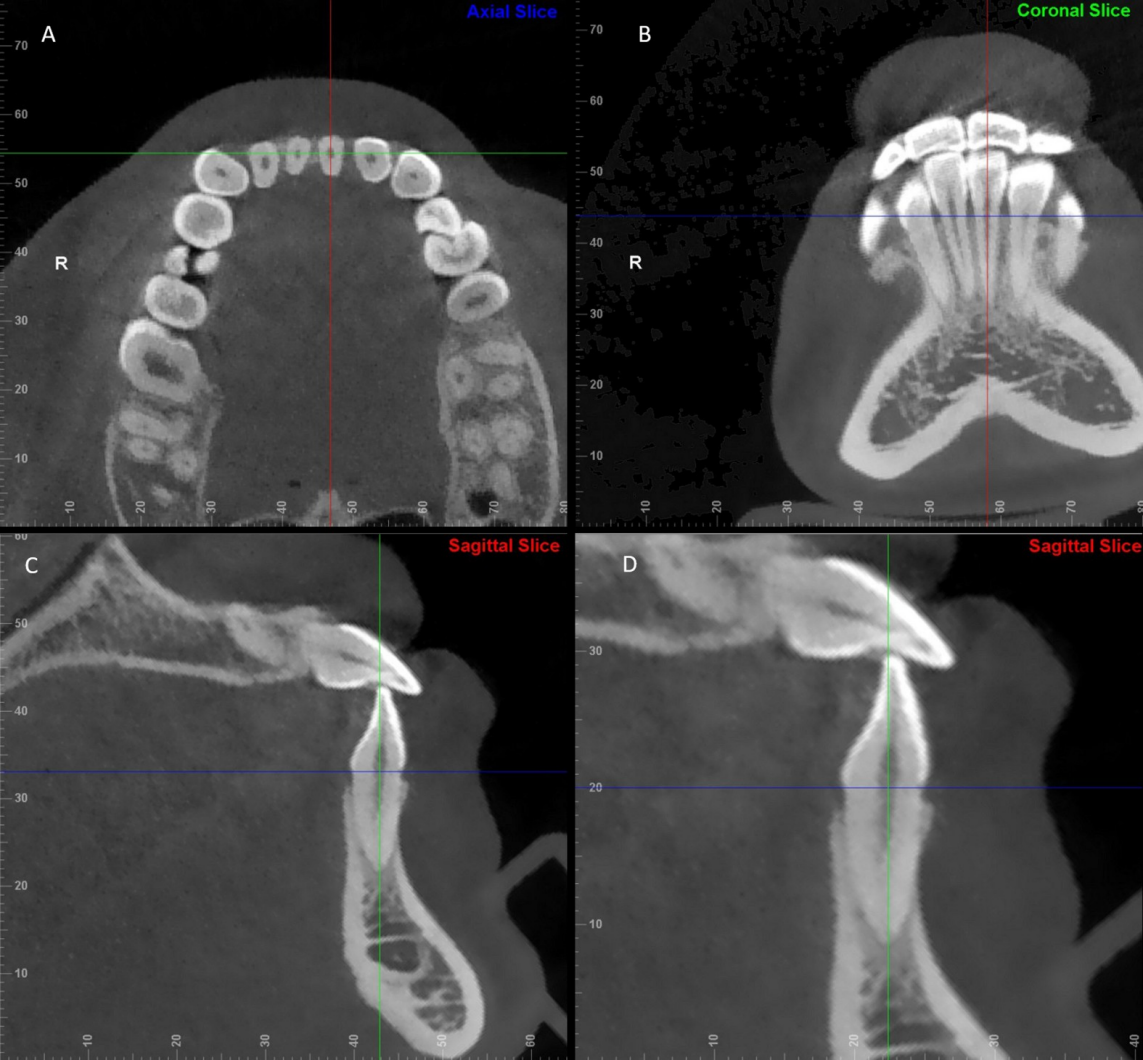
A detailed procedure for locating the measurement plane. Three guidelines with different colors representing the correlated planes as follows: blue, axial plane; red, sagittal plane; green, coronal plane. A, Adjust the location of the axial plane by passing the blue guideline through the CEJ of the selected anterior tooth in both the coronal and sagittal views; then, rotate the green guideline until the intersecting line is the shortest. B, Rotate the red guideline until it passes through the root apex and the midpoint of the incisal margin. C, Rotate the green guideline until it passes through the root apex and the midpoint of CEJ line. D, To ensure precise and accurate identification of the anatomic structures, the largest labiolingual section of the anterior tooth displayed in the corrected sagittal view was chosen as the measurement plane.

**Fig 2:**
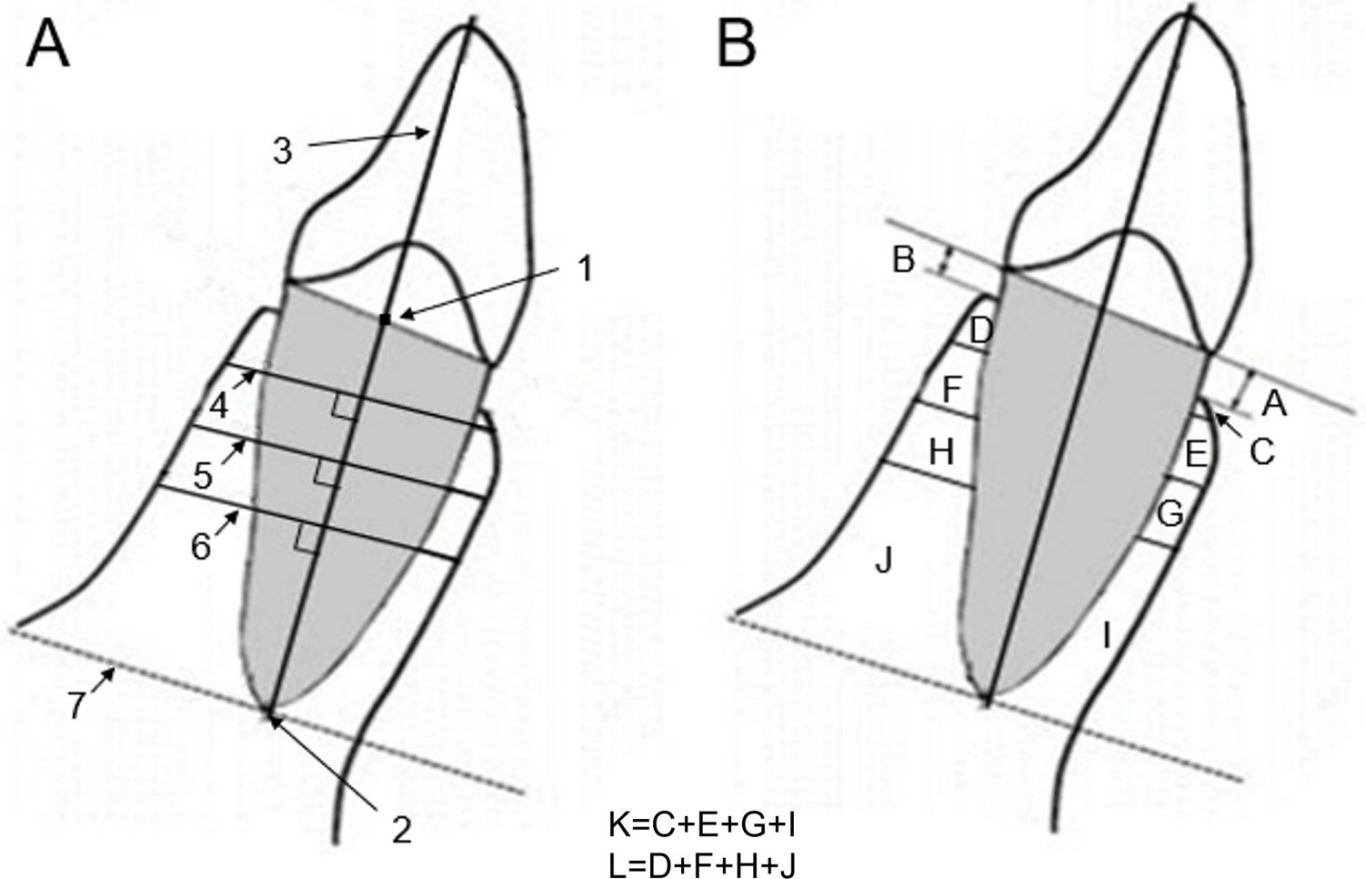
**(A) Landmarks and reference planes**. (1) midpoint of the CEJ line; (2) root apex (RA) point; (3) long axis (LA; a line from points 1 to 2); (4,5,6) intersecting line perpendicular to LA at the 2 mm, 4 mm, 6 mm below the CEJ line; (7) intersecting line perpendicular to LA at RA; **(B) Variables**: A and B, vertical alveolar bone level (distance from CEJ to alveolar crest parallel to LA); C and D, alveolar bone area (ABA) at 2 mm below the CEJ line; E and F, ABA at 4 mm below the CEJ line; G and H, ABA at 6 mm below the CEJ line; I and J, apical ABA; and K and L, total ABA on the labial (C+E + G + I) and palatal sides (D+F + H + J). Paired variables are the labial and palatal sides.

The largest labiolingual section was defined as the measurement plane. Fig 1 illustrates the detailed procedure for locating the measurement plane. Fig 2 gives the definitions of landmarks, reference planes and variables. All landmark identifications and measurements (Fig 2) were adopted from Hyo-Won Ahn et al [8]. For maxillary anterior teeth, landmarks and variables were the same as those of mandibular teeth. However, the three reference planes were different, and the intersecting line perpendicular to the long axis (LA) was at the 2 mm, 4 mm, 6 mm above the CEJ line. All measurements were made twice, 2 weeks apart, by the same operator, and the mean of the 2 values was used. To reduce fiuctuations in measurement accuracy in this study, one trained orthodontist(JM) made all the measurements and was blinded to participants’ information. Ten patients were selected randomly and measurements were repeated 2 weeks after the first measurement to assess the intra-operator error using paired samples t-test. Result revealed that the intra-operator error was not statistically significant, and nearly all the correlations were above 0.65. S1 Table shows the details information in supporting information.

## Statistical analysis

All statistical analyses were performed with the Statistical Package for the Social Sciences (version 17.0; SPSS, Chicago, IL, USA). The means and standard deviations were calculated. No statistically significant differences were found in the VBL, ABA and thickness of the alveolar bone at the same side of the same left and right tooth (P>0.05). Therefore, this study will combine statistics for the same site of the same left and right tooth. The results of Chi-square test showed that there was no statistically significant difference between male and female patients (χ^2^ = 0.072, P = 0.788), and the data were combined for further tests. An independent samples t-test was performed for statistical comparisons. The significance level was set at a 2-tailed P value of 0.05. Data analysis was conducted by one author(JHJ) blinded to all information of participants.

## Results

### The vertical alveolar bone level (VBL) difference

First, we compared the vertical alveolar bone level (VBL) of the upper and lower incisors between high-angle skeletal Class II and Class III malocclusion patients. Descriptive statistics and intergroup comparisons are shown in Tables 3 and 4. No statistically significant differences were found between the 2 groups, except for the labial VBL of the maxillary central and lateral incisor. In the maxillary central and lateral incisors, the labial VBL was smaller in patients in skeletal Class III than it was in patients in skeletal Class II (P<0.05). Similarly, a previous study found that the skeletal Class II group had a greater prevalence of fenestration than the skeletal Class I and the skeletal Class III group. Fenestrations were more prevalent in the maxilla [9].

**Table 3:** Comparison of maxillary anterior vertical bone level between high-angle skeletal Class II malocclusion patients and high-angle skeletal Class III malocclusion patients (mm).

Alveolar bone level	U1				P-value	U2				P-value
	II(N = 62)		III(N = 59)			II(N = 62)		III(N = 57)		
	Mean	SD	Mean	SD		Mean	SD	Mean	SD	
**Labial CEJ-AC**	1.82	0.88	1.56	0.56	0.045*	2.13	0.82	1.86	0.58	0.035*
**Lingual CEJ-AC**	1.37	0.57	1.50	0.47	0.152	1.50	0.67	1.43	0.50	0.513

Independent t-test was performed. SD indicates standard deviation. U1 represent maxillary central incisors, U2 represent maxillary lateral incisors

* P<0.05

**Table 4:** Comparison of mandibular anterior vertical bone level between high-angle skeletal Class II malocclusion patients and high-angle skeletal Class III malocclusion patients (mm).

Alveolar bone level	L1				P-value	L2				P-value
	II(N = 60)		III(N = 58)			II(N = 56)		III(N = 54)		
	Mean	SD	Mean	SD		Mean	SD	Mean	SD	
**Labial CEJ-AC**	1.76	0.72	1.86	0.56	0.401	1.77	0.81	1.88	0.66	0.426
**Lingual CEJ-AC**	2.09	0.65	1.97	0.61	0.300	2.05	0.67	1.93	0.78	0.365

Independent t-test was performed. SD indicates standard deviation. L1 represent mandibular central incisors, L2 represent mandibular lateral incisors; * P<0.05

### The alveolar bone area (ABA) difference

In a second step, we investigated the alveolar bone area (ABA) of upper and lower incisors between high-angle skeletal Class II malocclusion patients and high-angle skeletal Class III malocclusion patients (Tables 5 and 6, respectively). In the maxillary central incisors, the ABA was significantly thinner in the patients of skeletal Class II than it was in patients in skeletal Class III, especially in terms of the ABA at 4 mm and 6 mm above the CEJ level on the labial side (P<0.05). No statistically significant differences were found in the area 2 mm above the CEJ level and the apical ABA, or total ABA on the labial side between the 2 groups (P>0.05). On the lingual side, the ABA was significantly thinner in patients in skeletal Class III than it was in patients in skeletal Class II, especially in terms of the apical ABA and total ABA (P<0.05). No statistically significant differences were found in the area 2 mm, 4 mm, and 6 mm above the CEJ level between the 2 groups (P>0.05). In the area of maxillary lateral incisors, the ABA was significantly thinner in the patients in skeletal Class II than it was in patients in skeletal Class III, especially in terms of apical ABA and total ABA on the labial side (P<0.05). No statistically significant differences were found in the area 2 mm, 4 mm, and 6 mm above the CEJ level on the labial and lingual side and in the area of apical ABA, or total ABA on the lingual side between the 2 groups (P>0.05).

**Table 5:** Comparison of maxillary alveolar bone area between skeletal Class II malocclusion patients and skeletal Class III malocclusion patients (mm^2^).

ABA	U1				P-value	U2				P-value
	II(N = 62)		III(N = 59)			II(N = 62)		III(N = 57)		
	Mean	SD	Mean	SD		Mean	SD	Mean	SD	
**Labial side**										
**2mm (C)**	0.36	0.45	0.36	0.45	0.951	0.32	0.62	0.40	0.75	0.535
**4mm (E)**	1.65	0.86	2.00	0.90	0.028*	1.37	0.94	1.68	0.88	0.057
**6mm (G)**	1.91	0.78	2.24	0.72	0.018*	1.46	0.73	1.70	1.17	0.187
**Apical**	5.65	3.30	5.57	2.85	0.892	2.78	2.59	4.16	3.19	0.011*
**Total (K)**	9.58	4.09	10.20	3.62	0.373	5.93	3.62	7.94	4.23	0.006*
**Lingual side**										
**2mm (D)**	0.66	0.58	0.61	0.50	0.668	0.31	0.39	0.30	0.20	0.894
**4mm (F)**	2.69	0.98	2.55	0.81	0.386	1.70	0.98	1.92	0.70	0.154
**6mm (H)**	4.31	1.37	4.10	1.36	0.392	3.06	1.40	2.88	1.09	0.433
**Apical**	29.74	13.82	24.06	8.86	0.007*	22.64	10.83	20.88	9.43	0.345
**Total (L)**	37.40	15.18	31.33	8.98	0.008*	27.70	12.34	25.97	10.56	0.412

C and D, alveolar bone area (ABA) at 2 mm above the CEJ line; E and F, ABA at 4 mm above the CEJ line; G and H, ABA at 6 mm above the CEJ line; I and J, apical ABA; and K and L, total ABA on the labial (C+E + G + I) and palatal sides (D+F + H + J)

* P<0.05

**Table 6:** Comparison of mandibular alveolar bone area between skeletal Class II malocclusion patients and skeletal Class III malocclusion patients (mm^2^).

ABA	L1				P-value	L2				P-value
	II(N = 60)		III(N = 58)			II(N = 56)		III(N = 54)		
	Mean	SD	Mean	SD		Mean	SD	Mean	SD	
**Labial side**										
**2mm (C)**	0.36	0.68	0.24	0.33	0.213	0.28	0.47	0.23	0.29	0.526
**4mm (E)**	1.13	0.62	1.01	0.76	0.364	1.15	0.77	1.15	0.82	0.978
**6mm (G)**	1.05	0.68	0.94	0.67	0.372	0.91	0.65	0.84	0.71	0.641
**Apical**	4.83	2.65	3.11	2.20	0.000*	4.86	3.45	3.42	2.69	0.017*
**Total (K)**	7.38	3.38	5.30	2.53	0.000*	7.20	3.78	5.65	2.68	0.015*
**Lingual side**										
**2mm (D)**	0.04	0.11	0.04	0.07	0.847	0.06	0.15	0.06	0.13	0.941
**4mm (F)**	0.94	0.61	0.79	0.60	0.186	1.02	0.72	0.87	0.66	0.235
**6mm (H)**	2.05	0.86	1.25	0.89	0.000*	2.17	0.88	1.33	0.97	0.000*
**Apical**	10.89	3.77	8.98	5.43	0.028*	14.75	5.73	9.81	6.14	0.000*
**Total (L)**	13.91	4.59	11.06	6.34	0.006*	18.00	6.51	12.06	7.31	0.000*

C and D, alveolar bone area (ABA) at 2 mm below the CEJ line; E and F, ABA at 4 mm below the CEJ line; G and H, ABA at 6 mm below the CEJ line; I and J, apical ABA; and K and L, total ABA on the labial (C+E + G + I) and palatal sides (D+F + H + J)

* P<0.05

In the area of the lower anterior teeth, the ABA was significantly thinner in the patients of skeletal Class III than it was in patients in skeletal Class II, especially in terms of apical ABA, total ABA on the labial and lingual sides, and ABA at 6 mm below the CEJ level on the lingual side (P<0.05). No statistically significant differences were found in the area 2 mm or 4 mm below the CEJ level on the labial and lingual side, and in the area 6 mm below the CEJ level on the labial side between the 2 groups (P>0.05).

### Thickness difference of the alveolar bone

In the third step, we investigated the thickness of the alveolar bone of the upper and lower incisors between high-angle skeletal Class II malocclusion patients and high-angle skeletal Class III malocclusion patients. Tables 7 and 8 show the values for the thickness of the alveolar in high-angle skeletal Class II malocclusion patients and high-angle skeletal Class III malocclusion patients. In the maxillary central incisors, the thickness of alveolar bone was significantly thinner in the patients in skeletal Class II than it was in patients in skeletal Class III, especially regarding 6 mm above the CEJ level on the labial side (P<0.05). In the area of apical level on the lingual side, the alveolar bone thickness was significantly thinner in patients in skeletal Class III than it was in patients in skeletal Class II (P<0.05). No statistically significant differences were found in the other areas on the labial and lingual side between the 2 groups (P>0.05). In the maxillary lateral incisors, the alveolar bone thickness was significantly thinner in the patients in skeletal Class II than it was in patients in skeletal Class III, especially regarding the apical level on the labial side (P<0.05). In the area 2 mm above the CEJ level on the lingual side, the alveolar bone thickness was significantly thinner in patients in skeletal Class II than it was in patients in skeletal Class III (P<0.05). No statistically significant differences were found in the other areas on the labial and lingual side between the 2 groups (P>0.05).

**Table 7:** Comparison of the thickness of maxillary alveolar bone between skeletal Class II malocclusion patients and skeletal Class III malocclusion patients (mm).

Thickness of alveolar bone	U1				P-value	U2				P-value
	II(N = 62)		III(N = 59)			II(N = 62)		III(N = 57)		
	Mean	SD	Mean	SD		Mean	SD	Mean	SD	
**Labial side**										
**2mm**	0.61	0.56	0.68	0.63	0.502	0.42	0.55	0.56	0.56	0.163
**4mm**	1.07	0.43	1.20	0.45	0.088	0.92	0.41	1.04	0.48	0.166
**6mm**	0.96	0.34	1.11	0.36	0.021*	0.64	0.41	0.74	0.56	0.255
**Apical**	1.92	0.81	2.13	0.90	0.169	1.58	0.75	2.06	1.19	0.009*
**Lingual side**										
**2mm**	0.94	0.55	0.93	0.50	0.907	0.56	0.50	0.73	0.37	0.035*
**4mm**	1.88	0.54	1.83	0.61	0.670	1.30	0.60	1.28	0.44	0.891
**6mm**	2.73	0.90	2.64	0.88	0.608	1.89	0.89	1.81	0.73	0.597
**Apical**	7.84	2.07	7.14	1.40	0.028*	6.30	1.80	5.96	1.72	0.291

* P<0.05

**Table 8:** Comparison of the thickness of mandibular alveolar bone between skeletal Class II malocclusion patients and skeletal Class III malocclusion patients (mm).

Thickness of alveolar bone	L1				P-value	L2				P-value
	II(N = 60)		III(N = 58)			II(N = 56)		III(N = 54)		
	Mean	SD	Mean	SD		Mean	SD	Mean	SD	
**Labial side**										
**2mm**	0.48	0.45	0.44	0.42	0.690	0.47	0.43	0.46	0.38	0.860
**4mm**	0.63	0.35	0.57	0.40	0.425	0.63	0.38	0.62	0.44	0.847
**6mm**	0.47	0.33	0.40	0.29	0.268	0.31	0.31	0.31	0.28	0.979
**Apical**	2.66	0.86	2.18	0.82	0.003*	2.84	1.05	2.29	0.99	0.006*
**Lingual side**										
**2mm**	0.11	0.22	0.18	0.25	0.128	0.12	0.26	0.18	0.30	0.283
**4mm**	0.89	0.36	0.61	0.43	0.000*	0.99	0.46	0.69	0.46	0.001*
**6mm**	1.26	0.53	0.78	0.59	0.000*	1.39	0.63	0.85	0.60	0.000*
**Apical**	4.26	1.01	3.86	1.29	0.063	4.52	1.24	3.76	1.31	0.002*

* P<0.05

**Fig 3:**
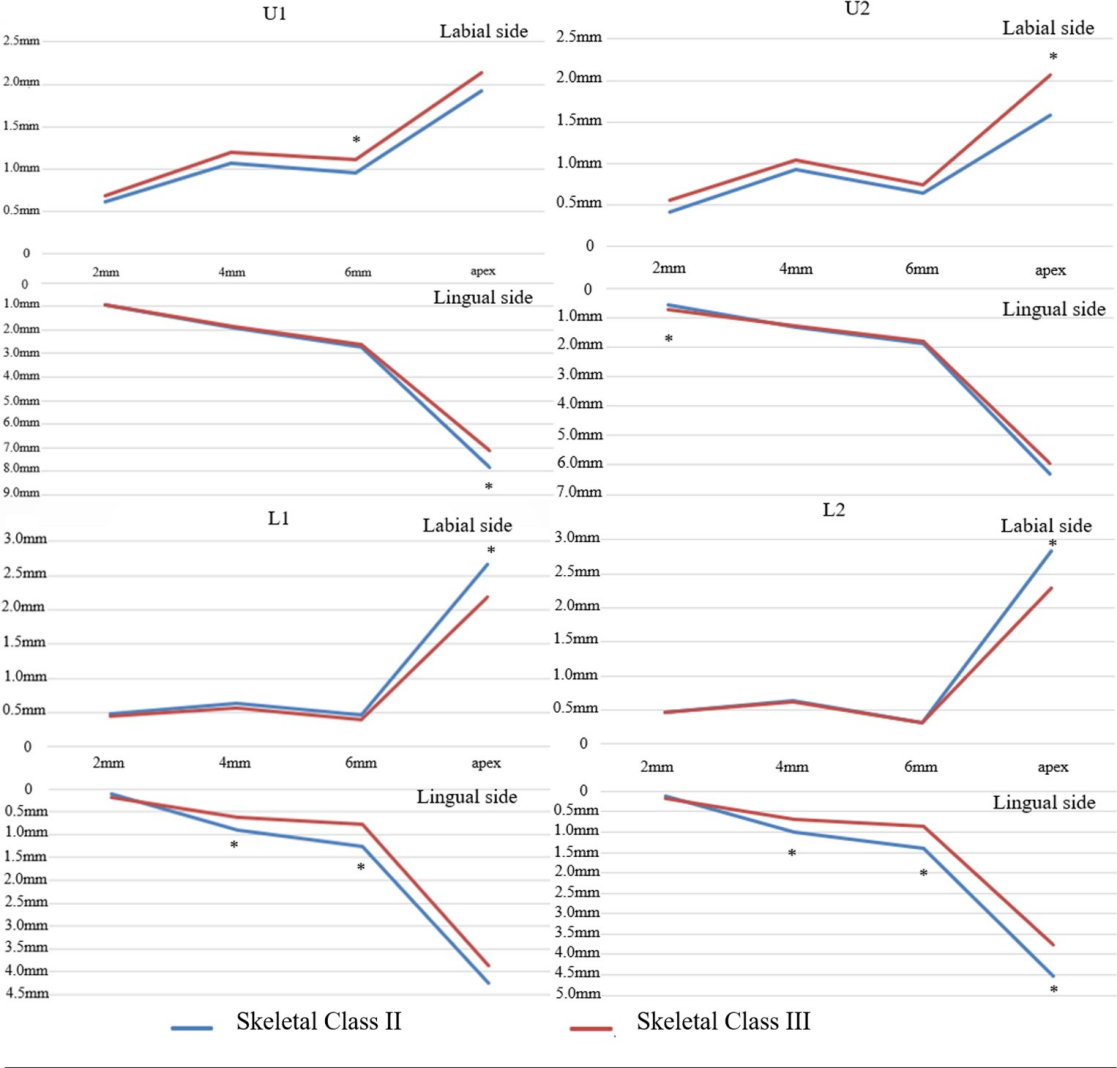
Comparison of the alveolar bone thickness of maxillary and mandibular anterior teeth between two groups. * P<0.05.

In the area of lower anterior teeth, the alveolar bone thickness of mandibular anterior teeth was significantly thinner in the patients in skeletal Class III than it was in patients in skeletal Class II, especially regarding the apical level on the labial side and at 4 mm and 6 mm below the CEJ level on the lingual side (P<0.05). In the area of the lateral incisor, the alveolar bone thickness was significantly thinner in patients in skeletal Class III than it was in patients in skeletal Class II, especially in the apical level on the lingual side (P<0.05). No statistically significant differences were found in the other areas on the labial and lingual side between the 2 groups (P>0.05). Fig 3 shows the alveolar bone mapping of maxillary and mandibular anterior teeth between the two groups.

## Discussion

Traditional two-dimensional images have inherent limitations, such as overlapping structure and geometric distortion, in the evaluation of bony architecture. The method is unable to accurately assess the condition of the alveolar bone before and after orthodontic treatment. With the advent of CBCT, visualization of the tooth and the bony anatomy has become possible because of the inherent accuracy of CBCT and the clipping function, which can accurately measure alveolar bone height and thickness [10]. CBCT has enabled alveolar bony measurement with good to excellent repeatability. Practitioners can repeatability select a stable cross section in accordance with the specific needs. In this study, we selected the median sagittal plane of a single tooth as an experimental section.

Ahn et al[8] found that the volume of alveolar bone significantly decreased on the palatal side during orthodontic treatment. Sadek et al [7] found that high-angle subjects can be at increased risk when moving incisors beyond alveolar bone support to reach significant antero-posterior incisor movement. So the decision as to how the bone may be affected by tooth movement is a critical consideration in treatment planning. Among the characteristics of facial morphology, facial type, such as short, average, and long, is an important factor in orthodontic treatment, mainly because facial type influences growth prediction of maxillofacial structures and goals of orthodontic treatment, as well as bite force and masticatory function [11–12]. During orthodontic treatment, we need to proclinate, retract or intrude or extrude anterior teeth. As the incisors move, the root of the teeth will gradually approach the cortex of the alveolar bone, which will limit further movement of the incisors. Edwards found that it does not appear possible to move the apex of the root more distally than the pretreatment position of the palatal plate. There appears to be an anatomic limitation to the distal movement of maxillary incisors [13].

Several studies provided evidence that a significant relationship exists between vertical facial type and the morphological features of the tooth-bearing region of the jaws. In previously cited research dealing with lower incisors, the alveolar bone was thicker in short-face type subjects than in long-face type patients [11–12]. Previous studies showed that the short-face type group showed a greater bone thickness in the anterior region of the maxilla and mandible than that of the long-face type group [14–16]. Baysal et al. [17] found that spongious bone was thinner, and the root apex was closer to the labial cortex in high-angle subgroups when compared with the Class II average-angle subgroup. Handelman [2] reported that labial and lingual alveolar widths were small in high-angle subjects as well as in Class III average-angle individuals. He believed that as the face lengthens, in part due to mandibular divergence, the incisors erupt to maintain overbite, and the alveolus becomes attenuated with thinning of the width between labial and lingual walls.

Other studies provided evidence that a significant relationship exists between sagittal facial type and the morphological features of the jaws. Baysal et al. [17] found that labial alveolar bone thickness of lower incisors was significantly higher in the Class I group compared with that of the Class II group. The bone level lingual to the mandibular incisor apex was narrower in the Class III group than in the Class I or Class II groups [2]. Kook [18] found that alveolar bone at the apex was significantly thinner in skeletal Class III malocclusion subjects than it was in normal occlusion subjects, except for the maxillary incisors. On the maxillary labial side, the mean value of alveolar bone thickness at the tooth apex showed no statistically significant differences between groups. However, on the maxillary lingual side, normal occlusion subjects showed wider bone thickness.

From previous studies, we knew that the morphological features of alveolar bone were affected by vertical facial type and sagittal facial type. To the authors’ knowledge, few studies have scientifically assessed the maxillary and mandibular alveolar bone status of high-angle adults with severe skeletal Class II malocclusions and high-angle adults with severe skeletal Class III malocclusions. Therefore, the purpose of this study was to use CBCT to evaluate the difference of alveolar bone of anterior teeth between high-angle adults with severe skeletal Class II malocclusions and high-angle adults presenting with severe skeletal Class III malocclusions. In our study, we found that the maxillary labial VBL was smaller in patients in skeletal Class III than it was in patients in skeletal Class II(P<0.05). This finding was similar to that of another study, where the skeletal Class II group had a greater prevalence of fenestration than did the skeletal Class I and skeletal Class III groups [9].

There were no statistically significant difference regarding apical ABA on the maxillary central incisors' labial side between the two groups (P>0.05). On the lingual side, the ABA was significantly thinner in patients in skeletal Class III than it was in patients in skeletal Class II, especially in terms of apical ABA and total ABA(P<0.05). In the maxillary central incisors, the alveolar bone thickness was significantly thinner in patients in skeletal Class III than it was in patients in skeletal Class II in the area of the apical level on the lingual side (P<0.05).This finding was similar to that of Kook’s study [18]. In the study of Eraydın F et[19] al, it was shown that alveolar bone thickness in Class III patients is relatively thin, which could be regarded as a risk factor for proclination. But our study revealed that around the maxillary central incisors, the ABA was significantly thinner in patients of severe skeletal Class II than that of severe skeletal Class III, especially in terms of the ABA at 4 mm and 6 mm above the CEJ level on the labial side (P<0.05).In the area of maxillary lateral incisors, ABA was significantly thinner in patients in skeletal Class II than it was in patients in skeletal Class III, especially in terms of apical ABA and total ABA on the labial side (P<0.05). The alveolar bone thickness of maxillary lateral incisors was also significantly thinner in patients in skeletal Class II than it was in patients in skeletal Class III, especially regarding the apical level on the labial side (P<0.05). These findings showed that the root of maxillary central and lateral incisors was placed more labially in patients in high-angle skeletal Class II than it was in patients in high-angle skeletal Class III, especially in the lateral incisors. As we know, the torque of the maxillary lateral incisor is smaller than that of the central incisor in MBT brackets. However, our study showed that maxillary lateral incisors were placed more labially than central incisors in patients in high-angle skeletal Class II. This finding suggested that the torque of maxillary lateral incisors should be increased in the subjects of high-angle skeletal Class II malocclusion.

Unlike Al Masri' s study[20], our study found that the apical ABA, total ABA of the mandibular alveolar bone in the area of the lower anterior teeth were significantly thinner in patients of skeletal Class III group than that of skeletal Class II group on both of the labial and lingual sides (P<0.05). The ABA at 6 mm below the CEJ level on the lingual side was significantly thinner in patients in skeletal Class III than it was in patients in skeletal Class II (P<0.05). The alveolar bone thickness of the mandibular anterior teeth was significantly thinner in patients in skeletal Class III than it was in patients in skeletal Class II, especially regarding the apical level on the labial side and at 4 mm and 6 mm below the CEJ level on the lingual side (P<0.05). In the mandibular lateral incisors, the alveolar bone thickness was significantly thinner in patients in skeletal Class III than it was in patients in skeletal Class II, especially regarding the apical level on the lingual side (P<0.05). These findings showed that the mandibular alveolar bone was thinner in patients in skeletal Class III than it was in patients in skeletal Class II, especially regarding the apical level and at 4 mm and 6 mm below the CEJ level on the lingual side.

## Conclusions

This cross-sectional study used CBCT to measure the difference in the alveolar bone of the anterior teeth. Our study found that the mandibular bony morphology in high-angle patients with skeletal Class III malocclusion was thinner than that in high-angle patients with skeletal Class II malocclusion. We also found that the root of maxillary central and lateral incisors was placed more labially in patients in high-angle skeletal Class II than it was in patients in high-angle skeletal Class III, especially in the lateral incisors.

## Supporting information

S1 Fig Reference planes on the lateral cephalogram. SN plane: the line between sella and nasion
MP plane: mandibular plane
ANB: subspinale-nasion-supramental angle
(The picture is drawn from "Johnston' Cephalometrics Handbook").
(TIF)

S1 Table Intra examiner correlation coefficient. (DOCX)
