# Supplementary figures and images for: Morphological analysis of the alveolar bone of the anterior teeth in severe high-angle skeletal Class II and Class III malocclusions assessed with cone-beam computed tomography
Source: PLoS One. 2019 Mar 25;14(3):e0210461. doi: 10.1371/journal.pone.0210461 (PMC6433292; doi:10.1371/journal.pone.0210461)

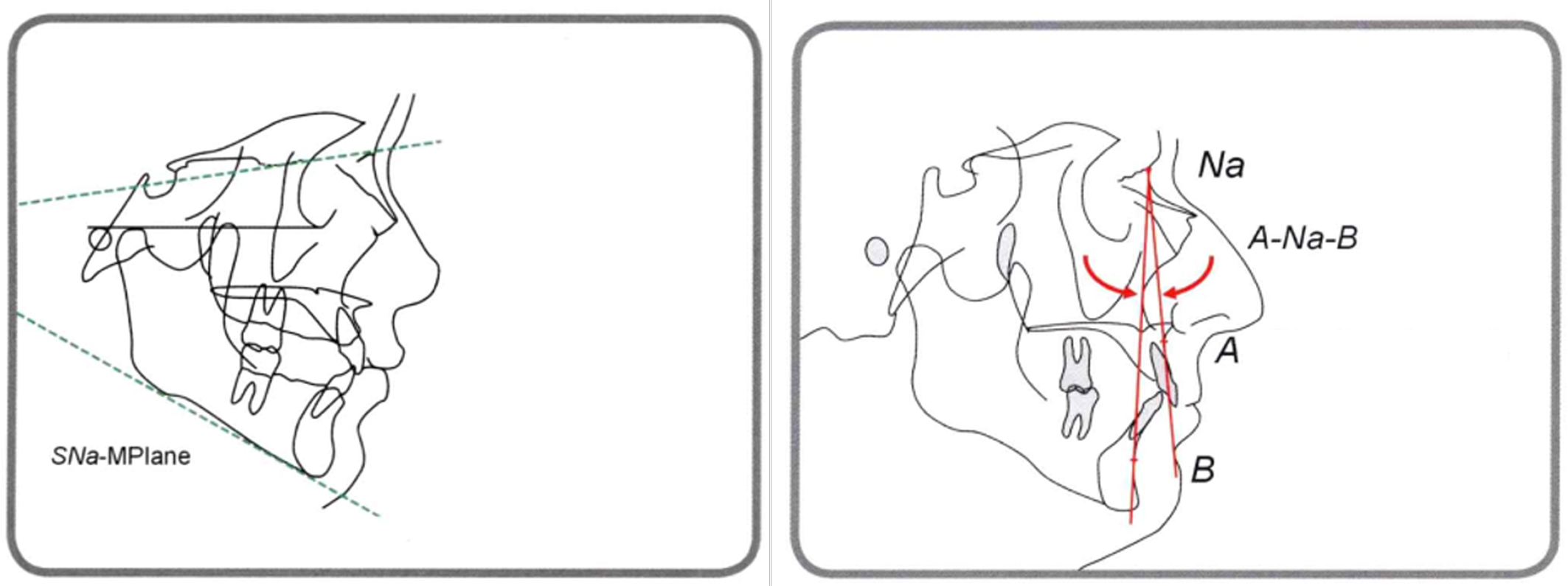

Supplement: S1 Fig — SN plane: the line between sella and nasion MP plane: mandibular plane ANB: subspinale-nasion-supramental angle (The picture is drawn from "Johnston' Cephalometrics Handbook"). (TIF) [file pone.0210461.s001.tif]
