# Supplementary material for: Morphological analysis of the alveolar bone of the anterior teeth in severe high-angle skeletal Class II and Class III malocclusions assessed with cone-beam computed tomography
Source: PLoS One. 2019 Mar 25;14(3):e0210461. doi: 10.1371/journal.pone.0210461 (PMC6433292; doi:10.1371/journal.pone.0210461)
Supplement: S1 Table — (DOCX) [file pone.0210461.s002.docx]

**S1 Table. Intra examiner correlation coefficient**

| Pairs | Correlations | Paired t-test | P |
| --- | --- | --- | --- |
| Labial CEJ-AC | 0.360 | 0.546 | 0.144 |
| Lingual CEJ-AC | 0.880 | 0.267 | 0.793 |
| Labial ABA(2mm) | 0.870 | 0.091 | 0.929 |
| Labial ABA(4mm) | 0.874 | 1.465 | 0.165 |
| Labial ABA(6mm) | 0.970 | 0.733 | 0.476 |
| Labial apical ABA | 0.908 | 1.598 | 0.132 |
| Labial total ABA | 0.943 | 1.178 | 0.258 |
| Lingual ABA(2mm) | 0.874 | 0.155 | 0.879 |
| Lingual ABA(4mm) | 0.923 | 0.193 | 0.165 |
| Lingual ABA(6mm) | 0.773 | 0.547 | 0.593 |
| Lingual apical ABA | 0.903 | 1.362 | 0.165 |
| Lingual total ABA | 0.934 | 0.701 | 0.495 |
| Labial thickness(2mm) | 0.939 | 0.985 | 0.341 |
| Labial thickness(4mm) | 0.851 | 0.525 | 0.608 |
| Labial thickness(6mm) | 0.890 | 1.003 | 0.333 |
| Labial thickness(apex) | 0.835 | 0.443 | 0.665 |
| Lingual thickness(2mm) | 0.971 | 1.060 | 0.307 |
| Lingual thickness(4mm) | 0.815 | 1.344 | 0.200 |
| Lingual thickness(6mm) | 0.887 | 0.384 | 0.707 |
| Lingual thickness(apex) | 0.909 | 0.503 | 0.623 |
